# Characterization of the chloroplast genome of *Calanthe henryi* (Epidendroideae; Orchidaceae)

**DOI:** 10.1080/23802359.2020.1770141

**Published:** 2020-06-02

**Authors:** Shu-Dong Zhang, Qin Wang, Mao-Mao Du, Li-Zhen Ling

**Affiliations:** School of Biological Sciences and Technology, Liupanshui Normal University, Liupanshui, China

**Keywords:** Chloroplast genome, phylogenetic analysis, *Calanthe henryi*, Epidendroideae, Orchidaceae

## Abstract

The first complete chloroplast (cp) genome sequences of an endemic and endangered species in China, *Calanthe henryi*, were reported in this study. The cp genome of *C. henryi* was 158,256 bp long, with two inverted repeat (IR) regions of 26,348 bp, a large single copy (LSC) region of 87,137 bp, and a small single copy (SSC) region of 18,423 bp. The cp genome of this species contained 113 genes, including 79 protein-coding genes, 4 ribosomal RNA genes, and 30 transfer RNA genes. The overall GC content was 36.7%. Phylogenetic analysis of 60 cp genomes within the subfamily of Epidendroideae suggests that *C. henryi* is closely related to *C. bicolor*.

*Calanthe henryi* Rolfe is a perennial plant in the subfamily of Epidendroideae (Orchidaceae) and usually grows in the evergreen forests from 1,600 m to 2,100 m. It is an endemic species in China and distributed in Hubei, Sichuan (Chen et al. 2009), Guizhou (Zhang 2007), Hunan (Yu et al. 2006) and Jiangxi (Xiao et al. 2017) provinces. At present, *C. henryi* has been regarded as the vulnerable endangered plant by IUCN (China Plant Specialist Group 2004). To promote the conservation of this species, we sequenced and analyzed the complete chloroplast (cp) genome of *C. henryi* using high-throughput sequencing technology.

The fresh leaf of *C. henryi* was collected from Yushe National Forest Park, Guizhou province, Southwest of China (N26°27′16″, E104°48′8″, 2,205 m). The specimen (lpssy0307) was deposited in the herbarium of the Liupanshui Normal University (LPSNU). Total genome DNA was extracted with CTAB method (Doyle and Doyle 1987), which was used for the library construction and sequencing on the Illumina HiSeq 2500 Platform. Approximately 2 Gb raw data were generated and used to *de novo* assemble the complete cp genome with SPAdes (Bankevich et al. 2012). All genes were annotated using PGA (Qu et al. 2019) with manual adjustment.

The cp genome of *C. henryi* (Genbank accession number MT385870) is a typical quadripartite structure with 158,256 bp long, including a pair of inverted repeat (IR, 26,348 bp) regions, a large single copy (LSC, 87,137 bp) region and a small single copy (SSC, 18,423 bp) region. The GC content of the cp DNA is 36.7%, which is similar to the other reported cp genomes from the genus of *Calanthe* (Dong et al. 2018; Miao et al. 2019; Zhong et al. 2019). A total of 113 unique genes were encoded, including 79 protein-coding (PCD) genes, 30 transfer RNA (tRNA) genes, and 4 ribosomal RNA (rRNA) genes. Of them, 7 PCDs (*ndhB*, *rps12*, *rpl23*, *rps7*, *rps12*, *rps19* and *ycf2*), 4 rRNAs (*rrn16*, *rrn23*, *rrn4.5* and *rrn5*), and 8 tRNAs (*trnA*-*UGC*, *trnH-GUG, trnl*-*CAU*, *trnI*-*GAU*, *trnL*-*CAA*, *trnN*-*GUU, trnR-ACG* and *trnV*-*GAC*) have two copies. Fifteen genes (*atpF*, *ndhA*, *ndhB*, *petB*, *petD*, *rpl12*, *rpl16*, *rpoC1*, *rps16*, *trnA-UGC*, *trnG-UCC*, *trnI-GAU*, *trnK-UUU*, *trnL-UAA* and *trnV-UAC*) contain one intron and three genes (*clpP*, *rps12* and *ycf3*) have two introns.

To determine the phylogenetic position of *C. henryi* within Epidendroideae, the complete cp genome sequences from 59 species of Epidendroideae and five species from Orchidoideae were downloaded from GenBank (Figure 1). All the complete cp genome sequences were aligned using MAFFT version 7.0 (Katoh and Standley 2013). Phylogenomic tree was reconstructed with the maximum likelihood (ML) and Bayesian inference (BI) methods (Ronquist et al. 2012; Stamatakis 2014). The ML and BI analyses generated the same tree topology (Figure 1). As shown in the phylogenetic tree (Figure 1), the species of *Calanthe* formed one monophyletic clade and *C. henryi* was close to *C. bicolor.* The *C. henryi* cp genome reported in this study may provide useful resources for the development of ornamental and ecological value as well as robust phylogenetic study at deep level of Orchidaceae in the future.

**Figure 1.**
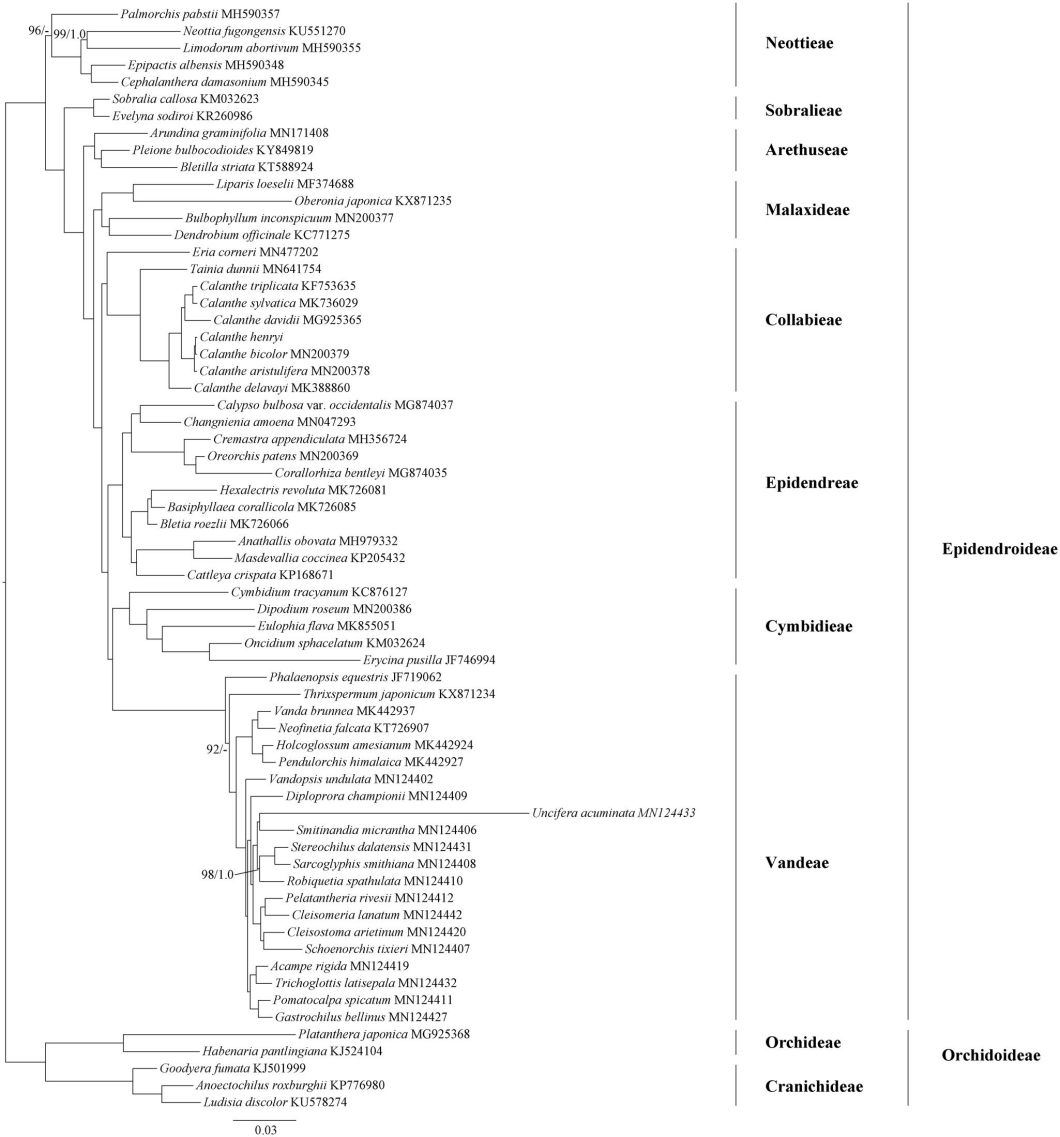
The maximum likelihood (ML) tree of Epidendroideae (Orchidaceae) inferred from the complete chloroplast genome sequences. ML bootstrap percentages (1,000 replicates) and Bayesian inference (BI) posterior probabilities are shown above clades if lower than 100 and 1.0 (dash indicate the brahches that are not supported by posterior probabilities).

## Data Availability

The data that support the findings of this study are openly available in National Center for Biotechnology Information at https://www.ncbi.nlm.nih.gov/, reference number MT385870. Submissions are accessioned but the sequence record is not examined and deposited by the GenBank annotation staff before it is free of errors or problems. Therefore, the live link to our dataset is not available at present.
